# Hepatitis C virus NS3‐4A inhibits the peroxisomal MAVS‐dependent antiviral signalling response

**DOI:** 10.1111/jcmm.12801

**Published:** 2016-02-10

**Authors:** Ana R. Ferreira, Ana C. Magalhães, Fátima Camões, Ana Gouveia, Marta Vieira, Jonathan C. Kagan, Daniela Ribeiro

**Affiliations:** ^1^ Department of Medical Sciences Institute for Biomedicine ‐iBiMED‐ and Department of Biology University of Aveiro Aveiro Portugal; ^2^ Division of Gastroenterology Boston Children's Hospital and Harvard Medical School Boston MA USA

**Keywords:** Hepatitis C virus, peroxisomes, MAVS, NS3‐4A

## Abstract

Hepatitis C virus (HCV) is the cause of one of the most prevalent viral infections worldwide. Upon infection, the HCV genome activates the RIG‐I‐MAVS signalling pathway leading to the production of direct antiviral effectors which prevent important steps in viral propagation.

MAVS localizes at peroxisomes and mitochondria and coordinate the activation of an effective antiviral response: peroxisomal MAVS is responsible for a rapid but short‐termed antiviral response, while the mitochondrial MAVS is associated with the activation of a stable response with delayed kinetics.

The HCV NS3‐4A protease was shown to specifically cleave the mitochondrial MAVS, inhibiting the downstream response.

In this study, we have analysed whether HCV NS3‐4A is also able to cleave the peroxisomal MAVS and whether this would have any effect on the cellular antiviral response. We show that NS3‐4A is indeed able to specifically cleave this protein and release it into the cytosol, a mechanism that seems to occur at a similar kinetic rate as the cleavage of the mitochondrial MAVS. Under these conditions, RIG‐I‐like receptor (RLR) signalling from peroxisomes is blocked and antiviral gene expression is inhibited. Our results also show that NS3‐4A is able to localize at peroxisomes in the absence of MAVS. However, mutation studies have shown that this localization pattern is preferred in the presence of a fully cleavable MAVS. These findings present evidence of a viral evasion strategy that disrupts RLR signalling on peroxisomes and provide an excellent example of how a single viral evasion strategy can block innate immune signalling from different organelles.

## Introduction

Hepatitis C virus (HCV) is a positive, single‐stranded RNA virus belonging to the *Flaviviridae* family. HCV infection is one of the most prevalent worldwide affecting 130–170 million individuals 1. With no effective vaccine, the current anti‐HCV therapies often lead to significant side effects and result in viral resistance 2.

Upon HCV infection, the virus is quickly sensed in the cytosol by soluble RNA helicases, RIG‐I and/or MDA5 3, 4, 5, which dimerize and interact with the mitochondrial antiviral signalling adaptor (MAVS) through their CARD domains 6, 7, 8, 9. This leads to a signalling cascade that culminates with the induction of interferons (IFN) and IFN‐stimulated genes (ISGs) that function as direct antiviral effectors, preventing important steps in viral propagation 10.

HCV has developed different mechanisms of evasion from the cellular immune response 11, 12, 13, 14, 15, 16. The viral serine protease NS3‐4A, besides being essential for HCV replication and assembling 17, is a key factor by which HCV is able to efficiently disrupt antiviral response 18. This complex is composed by two proteins: NS3, that contains the catalytic domain, and NS4A that acts as a cofactor stabilizing NS3 function and allowing the binding to organelle membranes 19, 20, 21. NS3‐4A is able to efficiently cleave MAVS at the mitochondria membrane, leading to the blockage of IFNs production 11.

Although initially assumed to localize exclusively at the mitochondria outer membrane 6, MAVS was also found to be present at peroxisomes 22, as well as at the mitochondria‐associated membranes (MAM) 23. The peroxisomal and mitochondrial MAVS‐dependent pathways result in different but complementing responses: the peroxisomal MAVS is associated to a rapid but short‐termed (type I IFN‐independent and type III IFN‐dependent) protection through the induction of ISGs, while the mitochondrial pathway leads to an type I IFN‐dependent production of ISGs, with delayed kinetics and amplifying the peroxisome‐dependent response 22.

Peroxisomes are ubiquitous and essential subcellular compartments with a critical role in a variety of metabolic processes 24, 25, 26, 27. The novel role as signalling platforms in antiviral defence underlies their importance in health and disease.

In this study, we have shown that HCV NS3‐4A is also able to cleave the peroxisomal MAVS and affect the cellular antiviral response.

## Results and discussion

### HCV NS3‐4A is able to specifically cleave the peroxisomal MAVS

**Figure 1:**
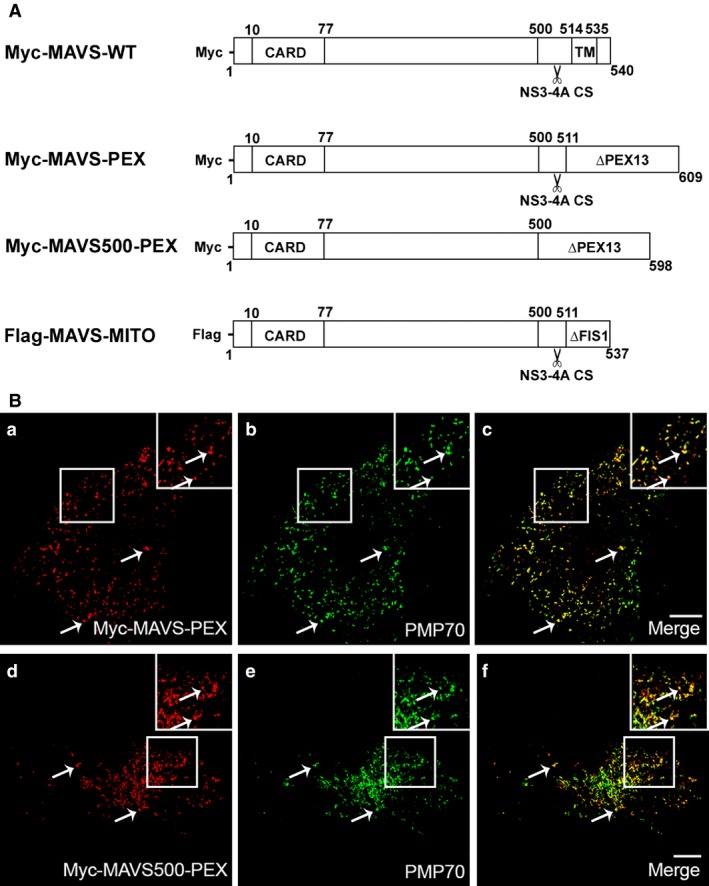
Localization pattern of the different peroxisomal MAVS used in this study. (**A**) Schematic representation of MAVS‐WT and mutant MAVS constructs used in this study. The cleavage site is represented by a scissors. (**B**) (a–c) MAVS‐PEX intracellular localization in Mefs MAVS‐KO cells (a) Myc‐MAVS‐PEX, (b) PMP70, (c) merge image of a and b. (d–f) MAVS500‐PEX intracellular localization in Mefs MAVS‐KO cells (d) Myc‐MAVS500‐PEX, (e) PMP70, (f) merge image of d and e. Arrows indicate co‐localization loci. Bars represent 10 μm. Representative images of three independent experiments.

Mitochondria and peroxisomes act in concert to establish the RIG‐I‐like receptor (RLR) dependent cellular response to viral infections 22, 23, 28. HCV NS3‐4A was shown to cleave the mitochondrial MAVS, leading to the dislocation of the N‐terminal fragment of MAVS from the mitochondria to the cytosol and inhibiting the MAVS‐dependent immune response 8, 11. Also the MAM‐localized MAVS was shown to be cleaved by this viral protein 23. To investigate whether NS3‐4A would also cleave the peroxisomal MAVS, we created a Myc‐tagged mutant of MAVS that localizes solely to peroxisomes, named Myc‐MAVS‐PEX (Fig. 1A). This construct was overexpressed in Mefs cells where MAVS had been previously knocked‐out (Mefs MAVS‐KO cells, described in 22) and, upon immunolocalization with antibodies against Myc and the peroxisomal marker PMP70, its peroxisomal localization was confirmed by confocal microscopy (Fig. 1B a–c). The specific cleavage site from MAVS that is recognized by NS3‐4A has been mapped to the Cys‐508 8, 11. We have also created a Myc‐tagged version of the peroxisomal MAVS that does not contain this cleavage site which we named Myc‐MAVS500‐PEX (Fig. 1A). This construct was similarly overexpressed in Mefs MAVS‐KO cells and its peroxisomal localization was confirmed by immunofluorescence (with antibodies against Myc and PMP70) and confocal microscopy (Fig. 1B d–f). Additionally, we have created a GFP‐tagged version of NS3‐4A (based on the construct described in 29), GFP‐NS3‐4A.

**Figure 2:**
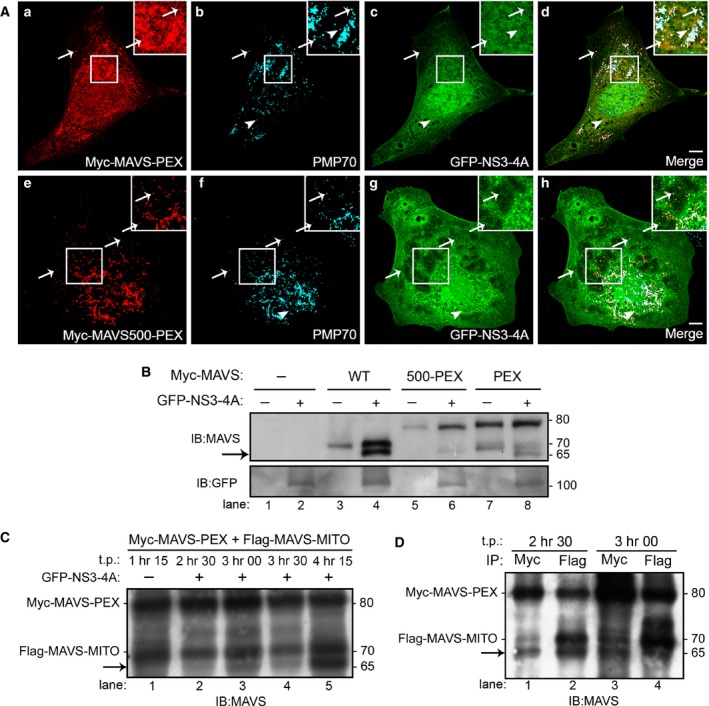
NS3‐4A cleaves the peroxisomal MAVS with similar kinetics as the mitochondrial MAVS. (**A**) (a–d) MAVS‐PEX is cleaved by NS3‐4A in Mefs MAVS‐KO cells. (a) Myc‐MAVS‐PEX, (b) PMP70, (c) GFP‐NS3‐4A, (d) merge image of a, b and c. (e‐h) MAVS500‐PEX is not cleaved by NS3‐4A (e) Myc‐MAVS500‐PEX, (f) PMP70, (g) GFP‐NS3‐4A, (h) merge image of e, f and g. Arrows indicate co‐localization loci between MAVS‐PEX or MAVS500‐PEX with peroxisomes and NS3‐4A. Full‐head arrows indicate co‐localization loci between peroxisomes and NS3‐4A. Bars represent 10 μm. (**B**) Western blot analysis of NS3‐4A cleavage of WT and mutant MAVS in Mefs MAVS‐KO cells. Arrow indicates the cleavage product of MAVS. (**C**) Time course of Myc‐MAVS‐PEX and Flag‐MAVS‐MITO cleavage by GFP‐NS3‐4A in Mefs MAVS‐KO cells. Arrow indicates the cleavage product of MAVS. (**D**) Pull‐down analysis of the Myc‐MAVS‐PEX and Flag‐MAVS‐MITO in Mefs MAVS‐KO cells. Immunoprecipitation was performed with antibodies against Myc and Flag. Arrow indicates the cleavage product of MAVS. Representative images of three independent experiments.

To analyse the possible cleavage of the peroxisomal MAVS by NS3‐4A, we cotransfected Myc‐MAVS‐PEX and GFP‐NS3‐4A in Mefs MAVS‐KO cells. As shown in Figure 2A a–d, in the presence of NS3‐4A, MAVS‐PEX localizes at the cytoplasm, confirming that NS3‐4A was able to traffic to peroxisomes and specifically cleave MAVS. As shown by the arrows in Figure 2A a–d, some MAVS‐PEX remains localized at the peroxisomes. This is because of the fact that, at the time‐point that the cells where collected (24 hrs post‐transfection) not all the MAVS had yet been cleaved. This is supported by the presence of both Myc‐MAVS‐PEX and GFP‐NS3‐4A at the same peroxisomes (as shown by the arrows in Fig. 2A a–d), where the cleavage has not yet occurred. It is important to notice the presence of NS3‐4A at some peroxisomes that do not contain MAVS‐PEX. Whether this represents NS3‐4A that remains attached to the peroxisomes after cleavage of MAVS‐PEX or NS3‐4A that simply migrated to peroxisomes that did not contain MAVS‐PEX will be further investigated and discussed below in this manuscript. As expected, Myc‐MAVS500‐PEX was not cleaved by GFP‐NS3‐4A (Fig. 2A e–h).

To confirm the cleavage of MAVS‐PEX by NS3‐4A, we performed Western blot analyses of lysates from Mefs MAVS‐KO cells expressing MAVS‐PEX, MAVS500‐PEX or wild‐type (WT) MAVS (described in 22) in the presence or absence of NS3‐4A. As shown in Figure 2B, a band corresponding to the expected size of the cleaved N‐terminal fragment of MAVS appears when either MAVS‐WT or MAVS‐PEX were cotransfected with NS3‐4A (indicated by the arrow, lanes 4 and 8), confirming that both proteins were cleaved by the viral protease.

### The cleavage of the peroxisomal and mitochondrial MAVS by NS3‐4A seems to occur with similar kinetics

The peroxisome‐dependent RLR response to viral infections occurs faster than the mitochondrial one, which is slower but long‐lasting, stabilizing the response initiated at peroxisomes 22. We wondered whether the virus would somehow kinetically distinguish MAVS targeting at these two different organelles, more specifically, whether NS3‐4A would cleave the peroxisomal MAVS faster than the mitochondrial one, to initially counteract the faster RLR response. To answer this question, we have constructed a mitochondria‐targeted version of MAVS (Flag‐MAVS‐MITO). The Myc‐MAVS‐PEX and Flag‐MAVS‐MITO were cotransfected into Mefs MAVS‐KO cells and the lysates were collected at different time‐points and analysed by Western blot. As shown in Figure 2C, as early as 2 hrs 30 min post‐transfection, it is already possible to observe a band that corresponds to the cleaved N‐terminal of MAVS (lane 2, indicated by the arrow). As it is not possible, with this experiment, to distinguish whether this band corresponds to the peroxisomal or mitochondrial MAVS, we have performed pull‐down analyses of these lysates with antibodies against Myc or Flag at the two lower time‐points where the cleavage was observed. As shown in Figure 2D, at 2 hrs 30 min both Myc‐tagged and Flag‐tagged N‐terminals of MAVS were pulled‐down, indicating that both peroxisomal and mitochondrial MAVS had already been partially cleaved. At these low post‐transfection time‐points, it is extremely difficult to obtain the necessary amount of protein to perform these analyses (cells are still recovering from the transfection procedure and the produced protein level in each cell is still low), which precluded the analyses at even lower time‐points. Our results show that at 2 hrs 30 min post‐transfection both MAVS are cleaved, but does not allow any specific conclusion concerning which one is the first to be cleaved. However, if at this time‐point we can already clearly observe this cleavage, it is tempting to extrapolate that NS3‐4A will cleave MAVS at both organelles with similar kinetics. Nevertheless, these results show that the virus does not preferentially cleave the mitochondrial MAVS, confirming the relevance of the peroxisome‐dependent pathway and the importance of its inhibition by HCV for viral propagation.

### NS3‐4A cleavage of peroxisomal MAVS strongly inhibits the peroxisome‐dependent antiviral cellular response

**Figure 3:**
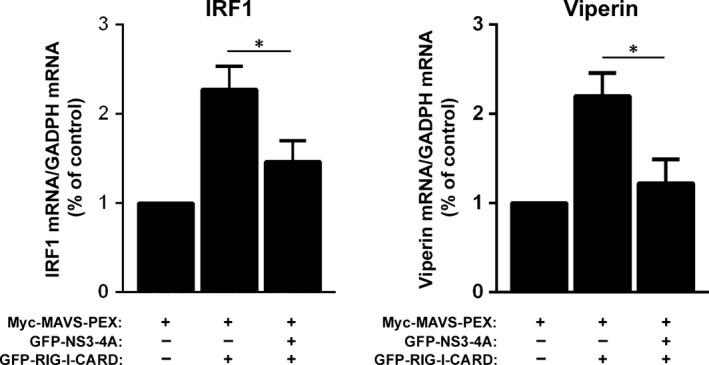
Cleavage of the peroxisomal MAVS by NS3‐4A inhibits the peroxisomal‐dependent production of antiviral compounds. RT‐qPCR analysis of IRF1 and viperin mRNA expression in Mefs MAVS‐KO cells expressing Myc‐MAVS‐PEX and stimulated with GFP‐RIG‐I‐CARD in the presence or absence of GFP‐NS3‐4A. GADPH was used as control. Data represent the means ± S.E.M. of three independent experiments. Error bars represent S.E.M.. *P < 0.05 in one‐way anova, with Bonferroni's post‐test, conditions were compared with the control Myc‐MAVS‐PEX condition.

To analyse whether the cleavage of the peroxisomal MAVS by NS3‐4A would cause the inhibition of the RLR signalling, we have co‐transfected Myc‐MAVS‐PEX and GFP‐NS3‐4A in Mefs MAVS‐KO cells. RLR‐dependent signalling events were stimulated in these cells by overexpressing a constitutively active version of RIG‐I (GFP‐RIG‐I‐CARD, 3). Twenty‐four hours after the co‐transfection, GFP‐RIG‐I‐CARD was transfected and, 6 hrs after, the production of mRNA of two ISGs (IRF1 and viperin) was quantified by RT‐qPCR. As shown in Figure 3, there was a clear increase on the production of IRF1 and viperin upon GFP‐RIG‐I‐CARD overexpression when compared with control cells solely transfected with Myc‐MAVS‐PEX. In the presence of NS3‐4A, however, the production of IRF1 and viperin decreased, clearly demonstrating that the cleavage of MAVS‐PEX by NS3‐4A disrupts MAVS signalling transduction from peroxisomes.

### NS3‐4A is able to traffic to peroxisomes in the absence of MAVS but preferentially targets this organelle in the presence of a fully cleavable version of this protein

The observation that NS3‐4A can be present at peroxisomes containing MAVS without the specific cleavage site (Fig. 2A), led us to wonder whether NS3‐4A would be attracted to this organelle by the specific presence of MAVS at its membranes or because of the specific characteristics or the organelle itself. Up to now, this study has never been performed for any cellular organelle where NS3‐4A is present.

**Figure 4:**
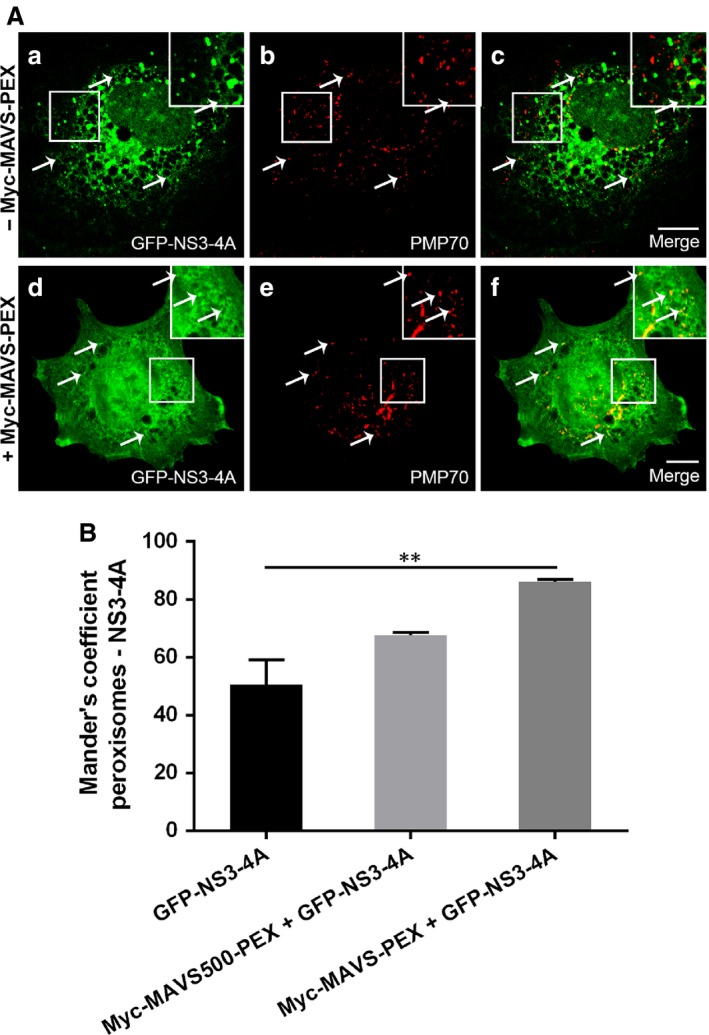
NS3‐4A intracellular localization analysis in Mefs MAVS‐KO cells. (**A**) (a–c) NS3‐4A intracellular localization in the absence of MAVS‐PEX (a) GFP‐NS3‐4A, (b) PMP70, (c) merge image of a and b. (d–f) NS3‐4A intracellular localization in the presence of MAVS‐PEX, (d) GFP‐NS3‐4A, (e) PMP70, (f) merge image of d and e. Arrows indicate co‐localization loci. Bars represent 10 μm. Representative images of three independent experiments. (**B**) Co‐localization between NS3‐4A and peroxisomes was analysed using Manders' coefficient. Data represent the means ± S.E.M. of three independent experiments, 45 cells were analysed for each condition. Error bars represent S.E.M.. **P < 0.01 in one‐way anova, with Bonferroni's post‐test.

To perform these analyses, we have expressed GFP‐NS3‐4A in Mefs MAVS‐KO cells, in the presence or absence of Myc‐MAVS‐PEX. As shown in Fig. 4A, even in the absence of the peroxisomal MAVS, the NS3‐4A can be partially found at peroxisomes. To statistically compare the level of NS3‐4A at peroxisomes in the presence or absence of MAVS at this organelle, we have analysed the co‐localization level in about 45 cells per condition (from three independent experiments). We have also compared our results with the level of co‐localization between NS3‐4A and peroxisomes in cells where the non‐cleavable Myc‐MAVS500‐PEX was present. Figure 4B shows a quite high co‐localization level between NS3‐4A and peroxisomes when no MAVS are present at the organelle. However, this level increases when peroxisomal MAVS are present, especially if these MAVS contain the cleavage site.

Previous studies have shown that NS4A, besides stabilizing NS3, is responsible for the localization of the NS3‐4A complex at the organelle's membranes 21, 30, 31. Tanji *et al*. 30 have shown that, in their system, in the absence of NS4A, more than 50% of the NS3 was localized in the cytosol fraction, while when coproduced with NS4A, most of it was found in the membrane fraction. On the other hand, one other study has shown that NS4A alone was not sufficient enough to confer the membrane association and stability of NS3 protein and have stressed the importance of the NS3 helix α0 for these processes 20. Nevertheless, up to now, no study has tackled the possibility that NS3‐4A would be attracted to the organelle's membranes by some specific membrane characteristics or by the presence of other (interacting) proteins in these membranes.

**Figure 5:**
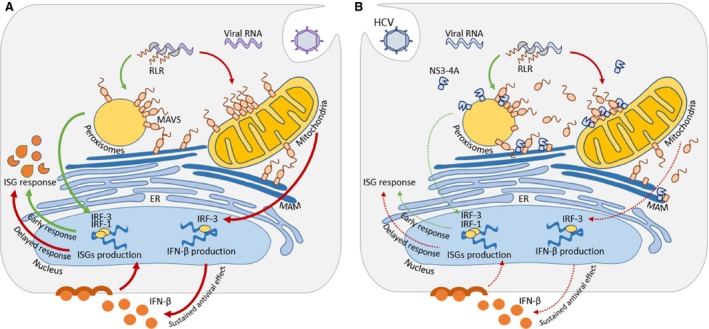
Model of organelle‐specific MAVS antiviral defence and HCV NS3‐4A effect. (**A**) During infection, viral RNA is released into the cytosol where it is recognized by RLR receptors. RLR receptors activate MAVS present at peroxisomes, mitochondria and MAM. Peroxisomal MAVS induces an early antiviral response, which is complemented by mitochondrial MAVS activation that mediates the expression of ISG through the secretion of type I IFN, promoting a delayed but sustained response. (**B**) HCV produces NS3‐4A, a non‐structural protein that, among other functions, allows evasion of the cellular antiviral defences. HCV NS3‐4A cleaves the adaptor protein MAVS at peroxisomes, mitochondria and MAM. MAVS cleavage leads to its release into the cytosol impairing the downstream signalling from peroxisomal and mitochondrial MAVS.

Our results suggest a model by which NS3‐4A traffics to peroxisomes by itself but remains longer at these organelle's membranes when it encounters its interacting partner MAVS (Fig. 5). The presence of NS3‐4A at the peroxisomal membranes seems to be even less transient when the MAVS possesses the original cleavage site. More studies should be performed to better dissect the NS3‐4A trafficking mechanisms as well as the characteristics of the organelle's membranes that are responsible for attracting this viral protein complex.

Our findings have not only uncovered an additional mechanism for HCV evasion from the host antiviral defences but also contribute to the unravelling of important antiviral signalling mechanisms that may affect many different viruses. Hence, these results may not only lead to the discovery of specific cellular targets for combat strategies against HCV, but also to the potential development of broad‐spectrum antiviral therapeutics.

## Materials and methods

### Plasmids and antibodies

Myc‐MAVS‐PEX was generated by replacing the previously described localization motif of MAVS 6 with the localization motif of the peroxisomal protein Pex13 32 and adding a Myc‐tag to the N‐terminal of the protein. This was performed with the MAVS‐WT and MAVS500‐PEX sequences (MAVS500‐PEX was based on the construct previously described by Dixit *et al*. (2010), where it was named MAVS‐PEX 22) as templates and cloning into the pCMV‐3C (Agilent Technologies, La Jolla, CA, USA) vector. Flag‐MAVS‐MITO was generated by replacing the Pex13 part of the Myc‐MAVS‐PEX construct by the localization motif of the protein Fis1 (as described in 22) as well as the Myc‐tag by a Flag‐tag. This was performed with the MAVS‐WT and MAVS500‐MITO sequences (MAVS500‐MITO was generated by cloning MAVS‐MITO, described in 22), as templates and it was cloned into the pCMV‐2A (Agilent Technologies, La Jolla, CA, USA) vector]. NS3‐4A was kindly provided by Dr. Eliane Meurs (Institut Pasteur, France), and it was cloned into a pEGFP‐C1 vector (Clontech Laboratories, Mountain View, CA, USA). GFP‐RIG‐I‐CARD was kindly provided by Dr. Weber (Philipps‐University Marburg, Germany).

In immunofluorescence, anti‐PMP70 (Sigma‐Aldrich, St. Louis, MO, USA) and anti‐Myc (71D10, Cell Signaling Technology, Beverly, MA, USA) were used to detect peroxisomes and Myc‐MAVS‐PEX respectively. In immunoblotting, anti‐MAVS (E‐3, Santa Cruz Biotechnology, Dallas, TX, USA), anti‐GFP (Invitrogen, Waltham, MA, USA), anti‐Myc (9E10, Santa Cruz Biotechnology, Dallas, TX, USA) and anti‐Flag (Sigma‐Aldrich, St. Louis, MO, USA) were used to detect MAVS, Myc‐MAVS‐PEX, Flag‐MAVS‐MITO respectively. Species‐specific anti‐IgG antibodies conjugated to HRP (BioRad Hercules, CA, USA) and the fluorophores TRITC (Jackson Immunoresearch, West Grove, PA, USA), Alexa 488 and Alexa 647 (both from Invitrogen, Waltham, MA, USA) were used as secondary antibodies.

### Cell culture and transfections

Mefs MAVS‐KO cells (described in 22) were cultured in Dulbecco's modified Eagle's medium supplemented with 100 U/ml, 100 mg/ml streptomycin and 10% foetal bovine serum (all from PAA Laboratories GmbH, Germany) and incubated at 37°C in atmosphere containing 5% CO_2_. These cells were transfected by microporation using the Neon Transfection System, under the manufacture recommendations (Invitrogen, Waltham, MA, USA). Cells were harvested and fixed from 2 hrs 30 min to 24 hrs after transfection.

### Immunofluorescence and microscopy analyses

Cells were processed for immunofluorescence as in 33 and photos were acquired with a Leica HCS A confocal microscope, using a Plan‐Apochromat 63× water objective (Leica Microsystems CMS GmbH, Mannheim, Germany), and a Zeiss LSM 510 confocal microscope, using a Plan‐Apochromat 63× and 100×/1.4 NA oil objectives (Carl Zeiss, Oberkochen, Germany). The lasers used were the 488 nm Argon‐ion laser, the 561 nm DPSS laser and the 642 nm HeNe. Digital images were optimized for contrast and brightness using Adobe Photoshop (Adobe Systems, San Jose, CA, USA). Co‐localization analysis was performed with the JACoP software (ImageJ, Bethesda, MD, USA) 34.

### Gel electrophoresis and immunobloting

Cells lysates and protein quantification were performed as in 33. After 15 min. incubation at 65°C, protein samples were separated by SDS‐PAGE in 7% polyacrylamide gels and transferred to a nitrocellulose membrane (PROTAN, Whatman, Dassel, Germany) by wet transfer. Immunoblots were treated with specific antibodies and enhanced chemiluminescence reagents (GE Healthcare, Waukesha, WI, USA).

### Immunoprecipitation

For immunoprecipitation of Myc‐MAVS‐PEX and Flag‐MAVS‐MITO, the Protein G Magnetic beads kit (Millipore, MA, USA) was used. At indicated time‐points, microporated cells were harvested and lysed as described. After protein quantification, protein samples were incubated with anti‐Myc (9E10, Santa Cruz Biotechnology, Dallas, TX, USA) and anti‐Flag (Sigma‐Aldrich, St. Louis, MO, USA) for 2 hrs at 4°C on a rotary mixer, before adding the beads to the mixture and incubating for 10 min. at room temperature. After washing, proteins were eluted in 3× laemmli sample buffer for 10 min. at 95°C. Immunoprecipitated samples were separated by running in a 7% SDS polyacrylamide gel.

### RNA extraction, cDNA synthesis and quantitative real‐time polymerase chain reaction

Total RNA was isolated using TriFast reagent (Peqlab, VWR International GmbH, Erlangen, Germany) and quantified using NanoDrop 1000 (Thermo Scientific, Waltham, MA, USA). cDNA synthesis was obtained using 2 μg RNA and M‐MuL V reverse transcriptase (New England Biolabs, Ipswich, MA, USA). RT‐qPCR was performed in duplicate using iTaq Universal SYBR Green Master Mix (BioRad, Hercules, CA, USA) and reactions were run on Applied Biosystems 7500 Real‐Time PCR System (Applied Biosystems, Walthman, MA, USA). Primer sequences used for quantification of IRF1 were 5′ GGTCAGGACTTGGATATGGAA 3′ and 5′ AGTGGTGCTATCTGGTATAATGT 3′, for viperin were 5′ TGTGAGCATAGTGAGCAATGG 3′ and 5′ TGTCGCAGGAGATAGCAAGA 3′ and for GAPDH were 5′ AGTATGTCGTGGAGTCTA 3′ and 5′ CAATCTTGAGTGAGTTGTC 3′, and were designed using the Beacon Designer 7 (Premier Biosoft, Palo Alto, CA, USA). GAPDH was used as a reference gene and data analysis was performed with the 2−ΔΔCT method.

### Statistical analyses

Statistical analysis was performed in Graph Pad Prism 5 (GraphPad Software, Inc., La Jolla, CA, USA). Data represent the mean ± standard error mean (S.E.M.). To determine the statistical significance between the experimental groups, the one‐way anova followed by Bonferroni's multiple comparison tests. *P* ≤ 0.05 was considered as significant.

## Conflict of interest

The authors declare that they have no conflict of interest.
